# Evaluation of the Dimensional Accuracy of 3D-Printed Anatomical Mandibular Models Using FFF, SLA, SLS, MJ, and BJ Printing Technology

**DOI:** 10.3390/jcm9030817

**Published:** 2020-03-17

**Authors:** Bilal Msallem, Neha Sharma, Shuaishuai Cao, Florian S. Halbeisen, Hans-Florian Zeilhofer, Florian M. Thieringer

**Affiliations:** 1 Clinic of Oral and Cranio-Maxillofacial Surgery, University Hospital Basel, CH-4031 Basel, Switzerland; neha.sharma@unibas.ch (N.S.); shuaishuai.cao@unibas.ch (S.C.); hans-florian.zeilhofer@usb.ch (H.-F.Z.); florian.thieringer@usb.ch (F.M.T.); 2 Medical Additive Manufacturing Research Group, Department of Biomedical Engineering, University of Basel, CH-4123 Allschwil, Switzerland; 3 Basel Institute for Clinical Epidemiology and Biostatistics, Department of Clinical Research, University Hospital Basel, University of Basel, CH-4031 Basel, Switzerland; floriansamuel.halbeisen@usb.ch

**Keywords:** 3D printing, additive manufacturing, binder jetting, dimensional accuracy, fused filament fabrication, mandible, material jetting, precision, RMS, selective laser sintering, stereolithography, trueness

## Abstract

With the rapid progression of additive manufacturing and the emergence of new 3D printing technologies, accuracy assessment is mostly being performed on isosymmetric-shaped test bodies. However, the accuracy of anatomic models can vary. The dimensional accuracy of root mean square values in terms of trueness and precision of 50 mandibular replicas, printed with five common printing technologies, were evaluated. The highest trueness was found for the selective laser sintering printer (0.11 ± 0.016 mm), followed by a binder jetting printer (0.14 ± 0.02 mm), and a fused filament fabrication printer (0.16 ± 0.009 mm). However, highest precision was identified for the fused filament fabrication printer (0.05 ± 0.005 mm) whereas other printers had marginally lower values. Despite the statistically significance (*p* < 0.001), these differences can be considered clinically insignificant. These findings demonstrate that all 3D printing technologies create models with satisfactory dimensional accuracy for surgical use. Since satisfactory results in terms of accuracy can be reached with most technologies, the choice should be more strongly based on the printing materials, the intended use, and the overall budget. The simplest printing technology (fused filament fabrication) always scored high and thus is a reliable choice for most purposes.

## 1. Introduction

Additive manufacturing (AM) is a rapidly evolving technology already used in various industries such as fashion, research and development, food, architecture, construction, aerospace, and healthcare. With the constant technical improvements in computer technology, the field of three-dimensional (3D) printing has simultaneously progressed. Especially in healthcare, various disciplines benefit from the new capabilities of computer-aided design (CAD)/computer-aided manufacturing (CAM) and 3D printing [1,2].

Several 3D printing technologies, such as fused filament fabrication (FFF), stereolithography (SLA), selective laser sintering (SLS), material jetting (MJ), and binder jetting (BJ), are used side by side in the healthcare sector. All these technologies have different advantages and limitations and therefore apply to different fields of medicine and dentistry. With the continuous development of each individual technology, none stand out significantly from the others and so all are represented in the daily medical routine.

Even though AM was introduced into medicine many years ago, the production of anatomical models often is still one of the main areas of application [3,4,5,6]. In opinion polls, surgeons still tend to regard anatomical models as advantageous for daily work [7]. For instance, anatomical models offer numerous advantages over other learning resources in understanding complex anatomical correlations [8]. The combination of optical and tactile sensitivity leads to a superior understanding and has defined the concept “touch to comprehend” [9]. A meta-analysis of 158 studies from 2005 to 2015 described further advantages such as possibilities for preoperative planning and time savings in the operating room, but emphasized that accuracy was not satisfactory in 34 studies [10]. Although most manufacturers provide the specifications in terms of accuracy, these are mostly uncertain in the final clinical application. In addition, most tests are performed on isosymmetric-shaped bodies [11], but the use of anatomical models may reveal larger dimensional errors. Measurements with skull and mandibular models revealed incorrect or completely missing anatomy [12]. Even deformations of 3D printed dental surgical guides were reported [13]. Inaccuracies in 3D printing applications can lead to inappropriate treatment that could harm the patient.

Identical procedures with the same material under the same circumstances do not necessary lead to identical results. Statistical standards described in the ISO 5725-1:1994/Cor 1:1998 subsumes the terms trueness and precision under the title accuracy [14]. Trueness refers to the closest results of the 3D printed models and the reference model, whereas the precision refers to the closest results under the different replicas by one printing technology. The main outcome for printing accuracy is the root mean square (RMS) value. The RMS is defined as the square root of the mean square (arithmetic mean of the squares of a group of values) between two parts.

The accuracy of an anatomical 3D model is affected by the sum of the errors that occur throughout the manufacturing process, starting with radiological imaging, image segmentation, standard tessellation language (STL) file generation, STL post-processing, slicing of the STL file for the printer control file, 3D printing, and post-processing. All these steps are heavily dependent on the machine, the software, and ultimately, the user. Studies have already confirmed the ability to create precise models for some of the technologies investigated, whereby data acquisition was mostly based on computed tomography (CT)/cone beam computed tomography (CBCT) or manual measurement procedures [15,16,17,18,19]. In particular, the digital imaging and communications in medicine (DICOM) to STL conversion process (image segmentation) can lead to widely different results. A recent study investigated the STL files converted from one DICOM data set by three different institutes, leading to different model volumes, model weights, and triangle counts [20]. Additionally, manual measurements are influenced by the variability of the operator and the difficulty in repeatedly selecting the exact landmark [19]. Although no measurement technique is error-free, computer-aided measurement methods can be beneficial here. Overall, these apparent differences can lead to inaccuracies and, as a result, can create false assumptions about the accuracy of a 3D printer.

In this study, the imaging and segmentation processes were skipped and the STL reference file was generated with a high-precision optical scanner. This resulted in a significant reduction of error sources that were not directly related to a 3D printer. However, certain influence of the high-precision scanner with its software and the 3D printer’s slicer software with its algorithms may remain. For this reason, most of the adjustable algorithms that could influence the scan file were disabled. We examined some of the most common 3D printing technologies using new digital analysis methods such as computer-aided measurement and optical scanning [2].

Hence, the aim of this study was to compare whether 3D printed mandibular models produced with several established and widely available 3D printing technologies are of comparable accuracy. The deviation patterns were analyzed with respect to trueness and precision. In addition, conclusions were drawn for everyday clinical practice and for each printing technology, and sources of error were discussed that could help practitioners select the appropriate technology that best suits their needs.

## 2. Materials and Methods

In the present study, a dry human bony mandible was examined and replicated 10 times by each of the five different 3D printers (*n* = 50). Every 3D printer included represents a different printing technology. After post-processing, the 50 replicas were also measured and compared with the reference data (*n* = 50) and with each other (*n* = 225). Statistical analysis was conducted to determine the closest results of the 3D printed models and the reference data (trueness) and the closest results under the different replicas by the according printing technology (precision). Ethical approval was not applicable.

### 2.1. Equipment and Materials

The 3D printers and material used for this study are summarized below (Table 1). A variety of additional devices were used for digitization and post-processing (Table 2).

### 2.2. Digitization of the Reference Model

**Figure 1:**
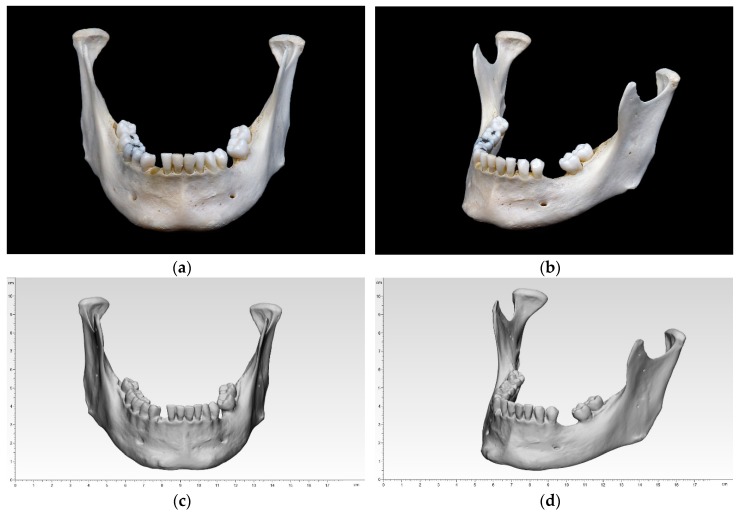
Bony mandibular reference: (**a**) front view; (**b**) side view; digitized mandibular (standard tessellation file (STL)) in 3-matic medical: (**c**) front view; (**d**) side view.

A dry human bony mandible was chosen as a reference model. This specimen reflects the most common characteristics of an adult mandible, e.g., recent loss of tooth #27 with unhealed extraction socket (most likely post mortem), bone resorption with healed extraction socket in the left mandible, remained wisdom tooth #32, as well as several tooth fillings. Three small markings were made on the ramus mandibulae on both sides, which should aid the digital alignment later on (Figure 1).

The specimen was registered by an optical white light desktop 3D scanner (EinScan-SP, SHINING 3D Tech. Co., Ltd., Hangzhou, China) with EinScan-S series software v. 2.7.0.6. The supplied manufacturer’s specifications included a point distance of 0.17–0.2 mm and a camera resolution of 1.31 megapixels. The digitization is performed with white light scanning technology and has a single shot accuracy of ≤0.05 mm. The following specifications were used: non-texture scan, bright, right camera on, and high dynamic range (HDR) off. The specimen was fixed to a turntable that rotated 8 times, every 45° until a 360° overview was achieved. This digitization was performed 4 times for upside, upside down, right side up, and left side up. The four digitizations were then merged into one digital model and an unwatertight model was created without any post-processing (no smoothing and no sharpness filter applied). The digitized data were exported in an STL file.

### 2.3. Preparation of STL File

The standard tessellation (STL) file of the reference model was imported into the corresponding 3D slicer software. This file contained the coordinates in a 3D grid. The software acted as a link between the planning data and the 3D printer. It sliced the digital design into cross sections, created a distinct path that the print head or laser could follow, and guided the subsequent printing [1]. Adjustments could be made to the print specification. After the print job was executed, the software generated a G-code that was sent digitally to the 3D printer and contained information about coordinates, nozzle temperature, and printing variables. The corresponding 3D slicer software are described separately in each of the following sections.

### 2.4. Three-Dimensional Printing of the Models (Replicas)

A key aspect that affects all printing technologies in the planning phase is the orientation of the print model. The print direction (xyz coordinate axis system) of the final model influences not only the printing time due to the number of layer lines, the strength due to the layer orientation, but also the accuracy due to error rates and support structures [21,22]. To leave the occlusal plane untouched for later analysis and to minimize the number of support structures required, all models were aligned vertically. Minor adjustments were made in the 3D slicer software before 3D printing. These adjustments ensured that the printing conditions corresponded to a clinical routine with a reasonable printing time and printing costs for a single model. According to the applied 3D printing technology, an adequate post-processing was conducted.

#### 2.4.1. Fused Filament Fabrication (FFF)

An Ultimaker 3 Ext. (Ultimaker B.V., Utrecht, The Netherlands) 3D printer was used to produce the 10 mandibular models with FFF technology. The chosen material was a white polylactic acid (Ultimaker PLA, Ultimaker B.V., Utrecht, The Netherlands) with a 0.4 mm print core AA nozzle. The software Cura v. 4.3.0 (Ultimaker B.V., Utrecht, The Netherlands) was adjusted to the standard print settings with a layer thickness of 150 microns and infill of 10%. The model was positioned in the center of the heated built platform. The printing time was 2 h and 40 min per model. Subsequently, post-processing was necessary to remove the supporting structures with fine cutting pliers.

#### 2.4.2. Stereolithography (SLA)

A Form 2 (Formlabs Inc., Somerville, MA, USA) 3D printer was used to produce the 10 mandibular models with SLA technology. The chosen material was a white resin White V4 (Formlabs Inc., Somerville, MA, USA). The software PreForm v. 3.0.1 (Formlabs Inc., Somerville, MA, USA) was adjusted to the standard printing settings with a layer thickness of 100 microns. The model was positioned in the center of the built platform. The printing time was 5 h and 16 min with approximately 816 layers. Subsequently, post-processing was necessary to remove the uncured resin from the print surface. This was achieved in a Form Wash (Formlabs Inc., Somerville, MA, USA) with a concentration of 90% isopropyl alcohol (IPA) bath for 10 min. To attain sufficient mechanical strength, an additional post-curing of 15 min was conducted in an UV Curing Apparatus (UVCA 2000; EnvisionTEC GmbH, Gladbeck, Germany) with heated, rotating UV radiation. Afterward, the supporting structures were removed with fine cutting pliers.

#### 2.4.3. Selective Laser Sintering (SLS)

An EOSINT P 385 (EOS GmbH, Krailling, Germany) 3D printer was used to produce the 10 mandibular models with SLS technology. The chosen material was a white polyamide 12 PA 2200 powder (EOS GmbH, Krailling, Germany). Due to the high acquisition costs of such a professional-grade 3D printer, a commercial 3D printing service (Composites Busch SA, Porrentruy, Switzerland) was commissioned. The printing time for each model was reported by the company as approximately 48 min.

#### 2.4.4. Material Jetting (MJ)

An Objet30 Prime (Stratasys, Ltd., Eden Prairie, MN, USA) 3D printer was used to produce the 10 mandibular models with MJ technology. The chosen materials were a white photopolymer resin VeroWhite (Stratasys, Ltd., Eden Prairie, MN, USA) and a water-soluble support material SUP706 (Stratasys, Ltd., Eden Prairie, MN, USA). The software Objet Studio Software v. 9.2.11.6825 (Stratasys, Ltd., Eden Prairie, MN, USA) was adjusted to the following printing settings with a tray material high-speed (HS), glossy surface option and a layer thickness of 28 microns. The model was positioned in the upper left corner of the built platform. The printing time was 12 h and 14 min. Subsequently, post-processing was required to remove the water-soluble supporting structures with a WaterJet Station (Stratasys, Ltd., Eden Prairie, MN, USA).

#### 2.4.5. Binder Jetting (BJ)

A ProJet CJP 660Pro (3D Systems, Inc., Rock Hill, SC, USA) 3D printer was used to produce the 10 mandibular models with BJ technology. The chosen materials were a VisiJet PXL Core (ZP151) as the core material and a VisiJet PXL Clear (ZB63) as the binder, both from 3D Systems, Inc. (Rock Hill, SC, USA). No coloring was applied. The 3DPrint Software v. 1.03 (3D Systems, Inc., Rock Hill, SC, USA) was adjusted to the standard print settings with a layer thickness of 100 microns. The printing time was 3 h and 22 min with approximately 665 layers. Subsequently, post-processing was required to remove unbound core material with a soft airbrush. The model could then be impregnated with acrylate or magnesium sulfate to improve its physical properties. However, since the manual application can lead to irregularities and deformations that influence the accuracy [17,18], this type of post-processing was omitted. The pure, airbrushed 3D model was used for the measurement.

### 2.5. Digitization of the 3D Printed Models (Replicas)

The digitization of the 50 3D printed mandibular replicas was conducted following the same workflow as described in Section 2.2.

### 2.6. Accuracy Analysis

**Figure 2:**
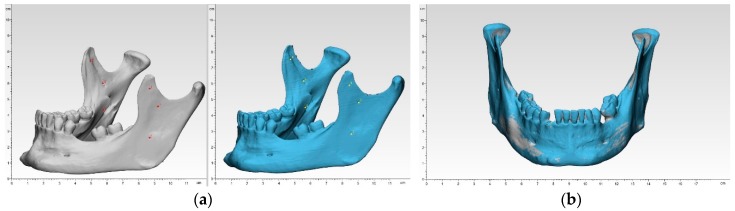
Registration process in 3-matic medical: (**a**) n-point registration; (**b**) after global registration.

To overcome the shortcomings of manual or automated coordinate-measuring in terms of operator variability and low number of landmarks, a digital measuring was chosen. Each of the 50 3D printed mandibular models were superimposed onto the reference mandibular model using a best-fit alignment method in the 3D analysis program 3-matic medical v. 12.0 (Materialise NV, Leuven, Belgium). All registrations were performed with an align feature by 6 manually placed control points in n-point registration. This function at first roughly overlay the two models. Here, the small markings in the ramus region were useful (Figure 2).

Then, a global registration was performed, which allows to modify the distance threshold, the number of iterations and the subsample ratio to ensure maximum possible superimposition. This semi-automatic algorithm for superimposition integrated in the software ensured an optimal alignment. After superimposition, a part comparison analysis was applied with a maximal tolerated deviation of ± 0.5 mm. The part comparison is based on point-based analysis algorithm (closest point) in the present study. For each test group, the positive and negative deviations were recorded and RMS values were calculated. To visualize the areas of aberrance where the target entity is located inside or outside the selected entity, a heat map was created.

For the trueness analyses, the reference model was compared with all replicas of the five different printing technologies (*n* = 50), and for the precision analyses all replicas of one printing technology were compared among each other (*n* = 225).

Since accuracy reflects systemic error, the RMS measures such bias. With an identical coordinate system between the datasets, the RMS was generated by the 3D analysis program. It describes the mean values of deviation between two data sets, typically of N-dimensional vector sets after superimposition. Consequently, a higher RMS value indicates a larger error, so the difference between reference and measurement data, and a smaller RMS value indicates less error.

### 2.7. Statistical Analysis

Descriptive statistics, including mean, standard deviation (SD), median, and first and third quartile ranges (IQR), and the root mean square (RMS) values, were collected for all 3D printed models from each 3D printer. To summarize the qualitative characteristics and to investigate the accuracy of the models, the RMS value was calculated for each set. The Shapiro–Wilk test was then applied to verify the distribution of the RMS values for each group of comparison. Either a one-way analysis of variance (ANOVA) with Tukey Kramer post-hoc pairwise test or a Kruskal–Wallis test with pairwise Wilcoxon rank sum (Mann–Whitney U) post-hoc tests adjusted for multiple-testing using a Holm–Bonferroni correction was used to identify intergroup differences in printing accuracy. All statistical analysis was performed using R statistical software (R Core Team 3.4.1, The R Foundation for Statistical Computing, Vienna, Austria).

## 3. Results

### 3.1. Qualitative Accuracy Assessment

**Figure 3:**
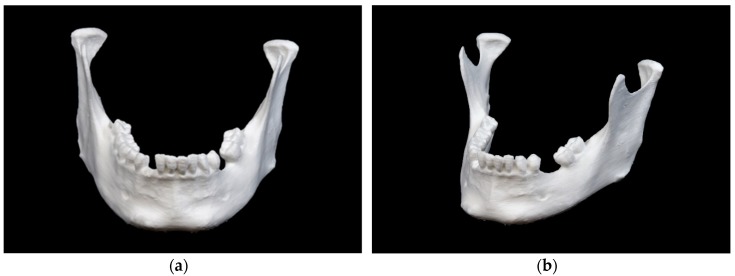
Mandibular model printed in fused filament fabrication (FFF) technology: (**a**) front view; (**b**) side view.

The mandibular model printed in FFF technology is show in Figure 3.

This model shows a rough surface with visible layer lines typical for this specific printing technique. The markings in the ramus region are visible, but quite blurred. In the mental region small remains of support structures can be observed.

**Figure 4:**
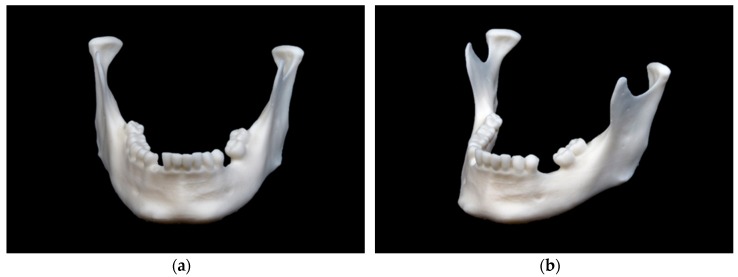
Mandibular model printed in stereolithography (SLA) technology: (**a**) front view; (**b**) side view.

The mandibular model printed using SLA technology is depicted in Figure 4.

This model shows a smooth surface without visible layer lines. Particularly in the tooth bearing section of the mandible, the smoothing is apparent, which leads to the interdental space being not adequately shaped. The markings in the ramus region are clearly visible and are not deflected by layer lines.

**Figure 5:**
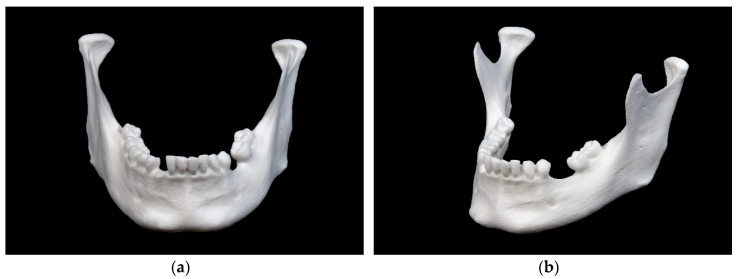
Mandibular model printed in selective laser sintering (SLS) technology: (**a**) front view; (**b**) side view.

The mandibular model printed in SLS technology is shown in Figure 5.

This model shows subtly recognizable layer lines and clearly visible markings in the ramus region.

**Figure 6:**
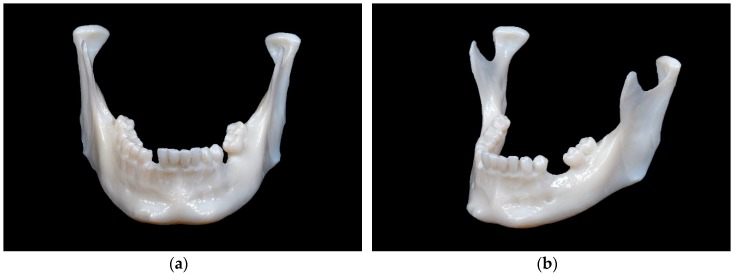
Mandibular model printed in material jetting (MJ) technology: (**a**) front view; (**b**) side view.

The mandibular model printed in MJ technology is depicted in Figure 6.

This model has a smooth and shiny surface because of its glossy finish. The surfaces that are not in contact with the support structure receive a glossy finish, whereas supported surfaces are matte. This leads to a non-uniform surface. Even if the markings in the ramus region are matte, they are clearly visible.

**Figure 7:**
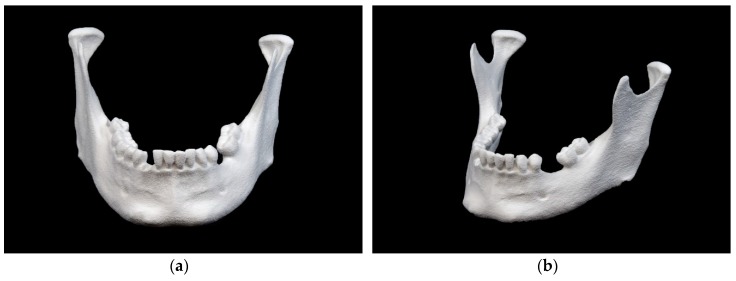
Mandibular model printed in binder jetting (BJ) technology: (**a**) front view; (**b**) side view.

The mandibular model printed in BJ technology is shown in Figure 7.

This model has a porous surface. The markings in the ramus region are recognizable, but not very clear due to the rough surface structure.

**Figure 8:**
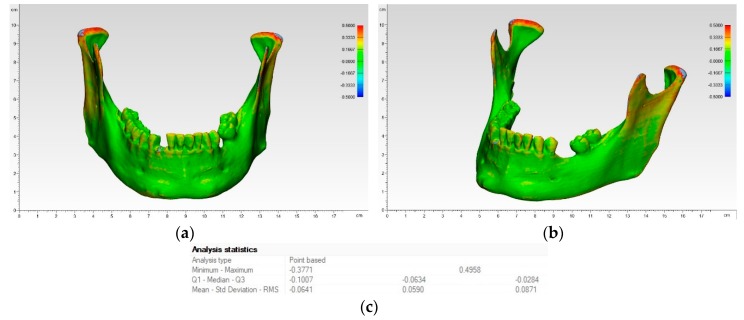
Heat map of mandibular model printed in SLS technology: (**a**) front view; (**b**) side view; (**c**) measurements.

The heat map of a mandibular model is provided in Figure 8.

In this heat map, the image of a mandible was divided into a grid by assigning each square a specific color according to its value. The blue areas indicate negative deviations, whereas red corresponds to positive deviations. A high degree of agreement between the values and thus low deviation from the object of comparison is shown in dark green color, which accounted for the largest part of the model. These are the more robust parts, like the mandibular corpus and mandibular angle. In contrast, the more delicate and sensitive areas, like the mandibular condyle as well as coronoid process, showed more light green to red nuances, which corresponds to positive deviations.

### 3.2. Quantitative Accuracy Assessment

#### 3.2.1. Trueness Analyses

Comparing all 3D printer types, the SLS printer (EOSINT P 385) appeared to have the highest overall trueness based on the RMS value (0.11 mm). The SLA printer (Form 2) had the worst overall trueness not only in terms of the RMS value (0.45 mm) but also in all other measurements (mean, SD, median, minimum, and maximum). The FFF printer (Ultimaker 3 Ext.) had the lowest mean and median values, but due to the higher variability in the other values (SD, minimum, and maximum), achieved a lower RMS value (0.16 mm) than the SLS printer. A summary of all statistics for trueness analysis of all 3D printer types are shown in Table 3.

**Figure 9:**
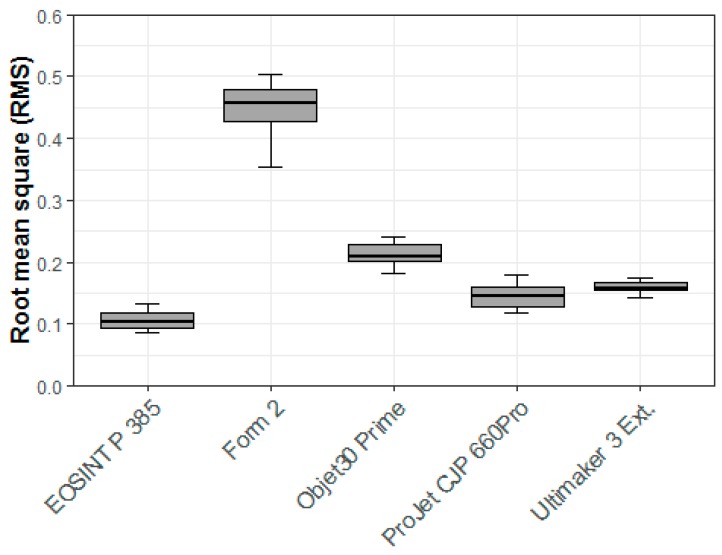
Box plot demonstrating trueness RMS (mm) values by 3D printer type.

The null hypothesis for the Shapiro–Wilk test assumes normally distributed data. The test yielded no significant results. This, combined with the inspection of the Q–Q (quantile–quantile) normal plots, implied that a parametric statistical test can be used. On this basis, an ANOVA with Tukey–Kramer post-hoc pairwise test was performed. The sensitivity analysis using the non-parametric Kruskal–Wallis rank sum test and Wilcoxon rank sum tests for the pairwise comparison showed similar results as the parametric analysis. The ANOVA analysis showed statistically significant differences in the trueness RMS values between the 3D printer types (*p* < 0.001). The pairwise comparison using the Tukey–Kramer post-hoc test revealed that all pairwise comparisons except the FFF printer (Ultimaker 3 Ext.) with the BJ printer (Projet CJP 660Pro) were statistically significant (Table 4). Combined with Table 5, we conclude that the SLS printer (EOSINT P 385) printer has the highest and the SLA printer (Form 2) has the lowest dimensional accuracy in terms of trueness (Table 5). The results are also shown graphically as box plots in Figure 9.

#### 3.2.2. Precision Analyses

Comparing all 3D printer types, the FFF printer (Ultimaker 3 Ext.) appeared to have the highest overall precision based on the RMS value (0.05 mm). The SLA printer (Form 2) had the worst overall precision in terms of the RMS value (0.09 mm). A summary of all statistics for precision analysis of all 3D printer types is provided in Table 6.

**Figure 10:**
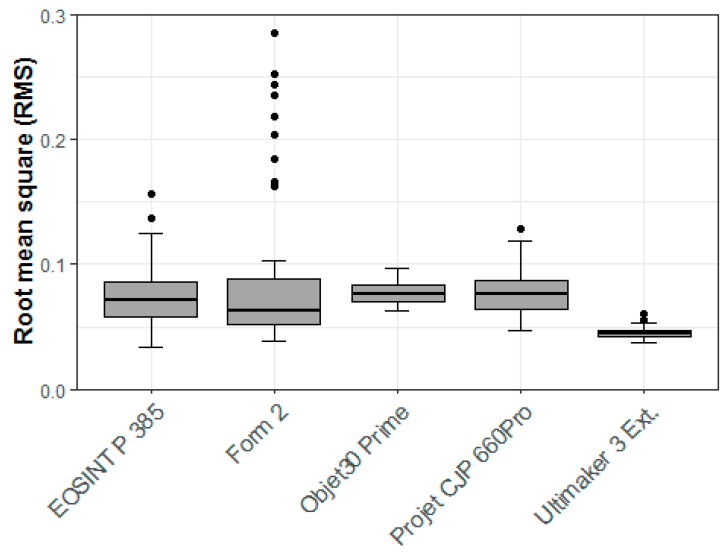
Box plot demonstrating precision RMS (mm) values by 3D printer type.

The Shapiro–Wilk test yielded several significant results. Combined with the inspection of the Q–Q normal plot, implied that a parametric statistical test could not be used. Based on this, the Kruskal–Wallis rank sum test with pairwise Wilcoxon rank sum tests were performed. The Kruskal–Wallis rank sum tests showed a statistically significant difference in precision RMS values between the 3D printer types (*p* < 0.001). The pairwise comparison using the Wilcoxon rank sum tests showed that after correction for multiple testing, only the pairwise comparisons of the FFF printer (Ultimaker 3 Ext.) were statistically significant (Table 7). We concluded that the FFF printer (Ultimater 3 Ext.) has the highest dimensional accuracy in terms of precision, whereas all other 3D printers perform similarly (Table 8). The results are also shown graphically as box plots in Figure 10.

## 4. Discussion

In the present study, the dimensional accuracies of anatomic mandibular models fabricated with five different 3D printing technologies were assessed and compared in terms of trueness and precision. Accuracy is one of the key aspects of medical 3D printing. Decisions made on the basis of inaccurate models can lead to wrong assumptions and, in the worst case, may harm the patient. Previous studies examined dimensional accuracy and expressed their results with absolute numbers in millimeters (e.g., mean absolute difference) or size ratios in percent (e.g., mean relative difference) [17,18,19,23]. However, deviations can occur as both positive and negative deviations from the reference object. An RMS value with the square of all values, then the average (mean) of these square values, and finally the square root of this mean value offers a new, and most likely more accurate, approach describing a deviation from the reference object. This study, like only few other recent studies mostly from the field of dentistry, focused on the RMS value [24].

Our initial focus was the trueness in terms of which 3D printer performs the closest when comparing RMS values between the STL data of the 3D printed models and the STL data of the dry human bony mandible. The highest overall trueness was achieved by the SLS printer (EOSINT P 385), followed by the BJ printer (Projet CJP 660Pro), the FFF printer (Ultimaker 3 Ext.), the MJ printer (Objet30 Prime), and finally the SLA printer (Form 2). The FFF printer (Ultimaker 3 Ext.) had the lowest mean and median values in the trueness analyses, but due higher variability in SD, minimum, and maximum, it did not perform as well in the overall RMS. This demonstrated that the mean and median values considered separately are not adequate measures of overall dimensional accuracy and highlights why our focus emphasized the RMS values for comparisons. The above results are consistent with the literature: SLS printers have greater dimensional accuracy than BJ printers [17,18]. Some authors obtained better results with an MJ printer than with a BJ printer [18], which was not the case here. This could have been caused by the post-processing of the BJ printer models [18]. To improve the mechanical properties, the plaster models are usually infiltrated with liquid cyanoacrylate, but since this is expected to lead to an uneven increase in size of the model due to manual application, and thus alter the model’s accuracy, this kind of post-processing was not applied in the present study.

The precision analyses which 3D printer produces the most uniform models when comparing the STL data of the 3D printed models of one single 3D printing technique to each other. The FFF printer (Ultimaker 3 Ext.) achieved the highest overall precision, followed by the SLS printer (EOSINT P 385), the MJ printer (Objet30 Prime), the BJ printer (Projet CJP 660Pro), and finally the SLA printer (Form 2). In this aspect, the most basic printing technology (fused filament fabrication) performed remarkably well and thus represents a reliable option for most medical applications. This result is consistent with that reported in the literature: entry-level 3D printers such as the FFF printer perform almost equally as good as professional devices such as the SLS printer in the field of medical-surgical applications [23]. The literature describes errors between 3D printed models and reference data that are generally less than 1 mm and typically less than 0.5 mm [25]. This is entirely in accordance with our results. Despite the statistically significance differences (*p* < 0.01), all differences in the printing technologies were very small and can be considered clinically insignificant for medical-surgical applications. However, the SLA printer (Form 2) did not perform as well as expected. SLA printers are generally characterized by their high accuracy. The reasons why this printer performed the worst among the 3D printers examined in this study are unclear and can only be suspected. One source of error could be the tray of the 3D printer. The polydimethylsiloxane (PDMS) layer may wear out after a few prints and may show signs of clouding, which can result in under-cured objects. In this study, however, a new tray was used to produce the 10 study models, which should have ensured proper fabrication. Another source of error could be the laser power, which may deplete with continuous usage. The special post-processing (IPA bath and UV post curing), which is fundamentally different from all others in this study, may have had an effect on the model. Unfortunately, this post-processing step could not be avoided when using SLA printers like the one for BJ printers because remaining resin residues on the surface may harm the operator and impact the measurements. The size and irregular geometry of anatomical models with varying layer thicknesses, angles, curvatures, and overhanging areas can possibly be altered by post curing, especially in previous under-cured models. However, no major dimensional inaccuracies were found in the preliminary tests of this 3D printer with isosymmetric-shaped test bodies. Since our measurements are in such contrast with those in the literature, a software problem or 3D printer malfunction cannot completely be ruled out and our values for the SLA printer should be considered with caution. To particularly evaluate this poor performance, follow-up studies are planned that will compare various SLA printers and their post-processing.

In view of the results of the accuracy analysis of printing technologies, a correlation with the purchase prices of the 3D printers can be observed. For example, in the ranking of trueness analysis, the SLS printer EOSINT P 385 is the most accurate and also has the highest purchase price of approximately $150,000, followed by the BJ printer ProJet CJP 660Pro with approximately $70,000, the FFF printer Ultimaker 3 Ext. with approximately $4000, the MJ printer Objet30 Prime with approximately $50,000, and finally the SLA printer Form 2 with approximately $3,500. With the exception of the FFF printer, which performs exceptionally well for its price, there is a correlation between the purchase price of these 3D printers and the accuracy. It should be noted that prices are estimates and may vary by reseller or country.

A basic knowledge of how 3D printing technologies work is essential to understanding possible sources of error. Fused filament fabrication (FFF) is a common and, in many ways, the simplest AM technology that performs material extrusion. The printer nozzle (extruder) loads the thermoplastic filament, melts it with the material’s specific temperature, and extrudes it. The extrusion head is attached to a two-axis system and extrudes the melted material along a previously determined path layer by layer while the platform drops. There are several 3D printing materials with different properties, e.g., acrylonitrile butadiene styrene (ABS), polylactic acid (PLA), and polyetheretherketone (PEEK). Advantages are the ease of use, the minimal troubleshooting, and low cost for common materials (except high-performance polymers like PEEK), making it the most affordable option. Limitations are the relative long printing time, the rather low resolution, the rough surface with visible layer lines, the need for post-processing (removing of support structures in case of mono-material print), poor mechanical properties due to layer lines, and warping. Layer lines occur because the melted thermoplastic material is slightly pressed against the previous layer, causing its surface to melt and bond to the new layer. These lines are visible depending on the layer thickness and tend to break up preferentially in case of mechanical stress (delamination). As the material hardens, their dimensions change, leading to force on the underlying layers. So, warping is also a common defect in FFF models. Due to the higher glass transition temperature and relatively high coefficient of thermal expansion, polymers like ABS are more sensitive to warping than PLA. To overcome this disadvantage and improve the bond of the object with the platform, especially when using materials with high melting temperatures, some 3D printers are equipped with a temperature-controlled heated build platform and chamber. The Ultimaker 3 Ext. uses a heated platform but no heated build chamber as standard. Like some other 3D printers, this can be upgraded into a closed system using a third-party expansion kit. Support structures lead to a lower surface quality and need to be removed after printing, thus harming the surface quality. Some printers offer the option to use dissolvable support structures, e.g., polyvinyl alcohol (PVA) that do not affect the surface quality like support material of the same material. The use of support structures makes the post-processing unfeasible in terms of sanding, polishing, priming, painting, or coating. According to the manufacturer’s specifications, the Ultimaker 3 Ext. with a 0.4 mm nozzle can achieve a layer thickness of 20–200 microns depending on the material used. An updated version of Ultimaker 3 Ext. is now available as the new Ultimaker 5 (Ultimaker B.V., Utrecht, The Netherlands).

Stereolithography (SLA) is a slightly more advanced AM technology that uses a vat of a liquid UV-curable photopolymer instead of an extrudable thermoplastic filament. It is regarded as the first 3D printing technology, which was invented in the early 1980s and later U.S. patented by C. W. Hull [26,27,28]. The 3D printer beams an ultraviolet (UV) laser beam to cure a photopolymer (acrylics) in a distinct previously determinate point, creating a solid layer. Then the build platform moves and a new layer will be created. After printing the model is cleaned in a chemical bath to remove resin residues and hardened by complete curing in an ultraviolet oven. Advantages are the in average high resolution and highly versatile material selection. Limitations are the high price especially for printing materials, the difficulty in handling liquid resin as it is sensitive to long exposure to UV light, rather poor mechanical properties due to brittle material and curling. It has isotropic mechanical properties because the resin won’t be fully cured at first and continues curing while post-processing. However, there are already numerous different materials available, with many different properties ranging from soft, transparent to very stable or even heat resistant. A technology limitation is the shrinking of the resin during curing, leading it to curling [11]. An important factor at bottom-up SLA printer, like the one in the present study, is the polydimethylsiloxane (PDMS) layer at the transparent bottom of the tray which wears out after some time. Additionally, the peeling step, the detachment of the resin from the bottom of the vat, forces an effect on the model. According to the manufacturer’s specifications, the Form 2 can achieve a layer thickness of 25–300 microns depending on the material used. As a successor to the Form 2, the new Form 3 (Formlabs Inc., Somerville, MA, USA), was recently launched on the market.

Selective laser sintering (SLS) represents another AM technology in which a laser fuses a polymer powder, creating a solid model. A CO_2_ laser melts thin layers of powder over a build platform creating one layer at a time as the platform slowly drops down and a roller creates another thin layer of powder. The unfused powder supports the model during the fabrication process and prevents the need for support structures. Since the whole model is embedded in powder, there is no need for support structures, allowing for complex geometric shapes. The advantages include the excellent mechanical properties that make it suitable for industrial applications, highly versatile material selection (nylon, polystyrene, metal), and no need for support structures. The limitations are the high price, being mainly affordable for professional 3D printing companies, and the fairly rough surface finish. The surface appears with porosity, which can lead to the absorption of water and thus affects the mechanical properties. Shrinkage and warping are the typical phenomena affecting dimensional accuracy. The shrinkage can be considered in the planning phase by increasing the model’s size. Particularly flat surfaces are affected by warping. Over-sintering can lead to melting of powder around the determined points, leading to loss of accuracy. According to the manufacturer’s specifications, the EOSINT P 385 can achieve a layer thickness of 100–150 microns depending on the material used.

Material jetting (MJ) is an AM technology in which a printhead dispenses droplets of a photosensitive liquid material (acrylic) that cures after ultraviolet (UV) light application, building a model layer by layer. Some printers allow the additional application of a dissolvable support structure. Advantages include high resolution and a unique surface finish. The surface can be either glossy or matte. Limitations include high costs, a long printing time, and poor mechanical properties. This AM technology is relatively expensive, making it impractical for basic applications. Since the layer thickness can be quite thin, the printing time is fairly long, so that in the present study, this AM technology took the longest to create a model. According to the manufacturer’s specifications, the Objet30 Prime can achieve a layer thickness of 16–28 microns depending on the material used.

Binder jetting (BJ) is an AM technology that selectively deposits binder onto a powder covered platform and bonds the material together layer by layer. A recoating blade applies a thin layer of powder over the build platform. Then, an inkjet nozzle selectively places droplets of a glue-like binding agent that bonds the powder particles together. Some printers can offer a full-color option. For colored models, the colored ink is included in this step. The material is most commonly sand, ceramics, or metal. After completing the whole layer, the platform is moved down, creating new space for a thin layer of powder, which is applied by the recoating blade. The same as with the SLS printer, the powder supports the model during the fabrication process, making support structures redundant. After printing, the model must remain embedded in the powder to fully cure. After removing the unbounded powder, which can be reused, the final model can be removed. Depending on the material, post-processing may vary. Plaster models have poor mechanical properties due to their high porosity, so can be infiltrated with acrylic or soaked with magnesium sulfate. The advantages of this 3D printing technique are that all the dimensional distortions that are connected to thermal effects as mentioned for previous techniques do not occur here and support structures are not required. Limitations include the usage of a liquid binder and the need for post-processing to enhance mechanical properties. When the liquid binder detaches from the nozzle, the ligament can break up and create small satellites. These satellites form a circular shape on impact. The increase in ink viscosity reduces such phenomena [29]. The impact of the droplets on the powder bed may lead to additional inaccuracies [30]. A study with anatomic models using BJ technology reported a mean absolute error of 0.32 mm (variance of 0.054 mm) for accuracy for structures above 10 mm in size [3]. According to the manufacturer’s specifications, the ProJet CJP 660Pro can achieve a layer thickness of 100 microns.

In view of the above-mentioned results of the accuracy measurements, it should be noted that the dimensional accuracy is determined by the xyz resolution of the three spatial dimensions. The xy plane describes the minimum feature size, while the resolution of the z axis describes the layer thickness. The minimum feature size is affected by the different 3D printing technologies, e.g., for FFF printers the flow dynamics of the extruded material are important, while for SLA, SLS, or MJ printers the specifications of the optical system and for BJ printers the infiltration of the adhesive in the powder. While for FFF printers it can be modified by changing the extruder diameter, it is usually fixed for MJ and BJ printers [25]. Reducing the layer thickness does not necessarily lead to more precise prints, as seen in the present study, but certainly to longer printing times as with the MJ printer. The disadvantage of increasing printing time can lead to an increased error rate, which also raises the probability of print failures. The layer thickness improves the transitions on the diagonals, but has little effect on vertical and horizontal edges. Therefore, a precise planning phase with regard to the geometry to be printed is required. Overall, the accuracy of a 3D printer is highly dependent on the AM technology, which also determines the printing materials to be used. The physical properties of the different printing materials affect the accuracy, e.g., different processing temperatures. As such, cooling and curing can lead to internal stresses and cause deformations. Our findings prove that unlike any other study with a wide range of 3D printing technologies and new digital measurement methods (like optical scans with respect to RMS values) and despite the ranking of printer accuracy, each 3D printer is highly accurate and therefore suitable for all medical–surgical applications. Despite the rapid developments in materials research, not every 3D printer is currently able to process the same materials, which represents the limiting factor in the field of application. Further studies should be conducted to determine whether the 3D printers evaluated here have similar accuracy values for differently sized models with similar materials. For instance, the size of the objects may influence the accuracy of certain AM technologies. Additionally, standardized methods for the examination of 3D printed models in healthcare with state-of-the-art digital measurement techniques should be established.

## 5. Conclusions

The findings here showed that all of the five evaluated printing technologies are very accurate. The SLS printer has the highest overall trueness (RMS 0.11 ± 0.016 mm), whereas the FFF printer has the highest overall precision (RMS 0.05 ± 0.005 mm). Despite the existing statistical significance of the differences in accuracy between all the 3D printers studied, when regarded separately, all are minor and acceptable for medical–surgical applications. However, the used printing material determines the field of application. Therefore, when choosing a 3D printer, the focus should no longer be primarily on the technology used, but rather on the desired application depending on the printing materials in relation to the total budget available. Therefore, practitioners should be aware of the practicability of each technology with its advantages and limitations.

A broad range of cost-effective in-house desktop 3D printers based on simple AM technologies, e.g., FFF technology, offer both high accuracy and the ability to process a wide range of different printing materials, including a growing number of biocompatible materials. This qualifies them for most medical–surgical applications, such as the fabrication of anatomical models for the pre-bending of osteosynthesis plates, for surgical drilling and cutting guides, for implants, or simply for educational purposes.

## Figures and Tables

**Table 1:** Three-dimensional (3D) printers, manufacturers, technology, and material

3D Printer	Manufacturer	Technology	Material
EOSINT P 385	EOS GmbH, Krailling, Germany	SLS ^1^	PA 2200
Form 2	Formlabs Inc., Somerville, MA, USA	SLA ^2^	White V4
Objet30 Prime	Stratasys, Ltd., Eden Prairie, MN, USA	MJ ^3^	VeroWhite SUP706
ProJet CJP 660Pro	3D Systems, Inc., Rock Hill, SC, USA	BJ ^4^	VisiJet PXL Clear (ZB63) VisiJet PXL Core (ZP151)
Ultimaker 3 Ext.	Ultimaker B.V., Utrecht, The Netherlands	FFF ^5^	PLA

^1^ selective laser sintering, ^2^ stereolithography, ^3^ material jetting, ^4^ binder jetting, ^5^ fused filament fabrication.

**Table 2:** Additional devices and manufactures.

Device	Manufacturer
EinScan-SP	SHINING 3D Tech. Co., Ltd., Hangzhou, China
Form Wash	Formlabs Inc., Somerville, MA, USA
UV Curing Apparatus (UVCA 2000)	EnvisionTEC GmbH, Gladbeck, Germany
WaterJet Station	Stratasys, Ltd., Eden Prairie, MN, USA

**Table 3:** Summary of all trueness analyses values (mm) for each 3D printer.

3D Printer	RMS ^1^	Mean	SD ^2^	Median	Minimum	Maximum
EOSINT P 385	0.11	−0.07	0.08	−0.06	−0.51	0.87
Form 2	0.45	0.23	0.39	0.17	−1.91	1.69
Objet30 Prime	0.21	0.17	0.13	0.15	−0.52	1.12
ProJet CJP 660Pro	0.14	0.09	0.11	0.08	−0.65	1.51
Ultimaker 3 Ext.	0.16	−0.01	0.16	0	−1.02	1.08

^1^ root mean square, ^2^ standard deviation.

**Table 4:** *p*-values of the Tukey–Kramer post-hoc test.

3D Printer	EOSINT P 385	Form 2	Object30 Prime	ProJet CJP 660Pro
Form 2	<0.01			
Objet30 Prime	<0.01	<0.01		
ProJet CJP 660Pro	0.01	<0.01	<0.01	
Ultimaker 3 Ext.	<0.01	<0.01	<0.01	0.6

**Table 5:** Summary of the trueness RMS values (mm) for each 3D printer type.

3D Printer	Mean ± SD ^1^	Median (Q1 to Q3)
EOSINT P 385	0.11 ± 0.016	0.10 (0.09 to 0.12)
Form 2	0.45 ± 0.044	0.46 (0.43 to 0.48)
Objet30 Prime	0.21 ± 0.02	0.21 (0.2 to 0.23)
ProJet CJP 660Pro	0.14 ± 0.02	0.15 (0.13 to 0.16)
Ultimaker 3 Ext.	0.16 ± 0.009	0.16 (0.16 to 0.17)

^1^ standard deviation.

**Table 6:** Summary of all precision analyses values (mm) for each 3D printer.

3D Printer	RMS ^1^	Mean	SD ^2^	Median	Minimum	Maximum
EOSINT P 385	0.07	0	0.17	0	−1.30	1.18
Form 2	0.09	0.01	0.24	0	−1.73	1.67
Objet30 Prime	0.08	0	0.16	0	−1.53	1.45
ProJet CJP 660Pro	0.08	0.01	0.16	0.01	−1.31	1.45
Ultimaker 3 Ext.	0.05	0	0.09	0	−1.29	1.21

^1^ root mean square, ^2^ standard deviation.

**Table 7:** *p*-values of pairwise Wilcoxon rank sum tests. ^1^

3D Printer	EOSINT P 385	Form 2	Objet30 Prime	ProJet CJP 660Pro
Form 2	0.918			
Objet30 Prime	0.345	0.056		
ProJet CJP 660Pro	0.918	0.35	0.918	
Ultimaker 3 Ext.	<0.01	<0.01	<0.01	<0.01

^1^ Adjustments for multiple testing using a Holm–Bonferroni correction were made.

**Table 8:** Summary of the precision RMS values (mm) for each 3D printer type.

3D Printer	Mean ± SD ^1^	Median (Q1 to Q3)
EOSINT P 385	0.07 ± 0.027	0.07 (0.06 to 0.09)
Ultimaker 3 Ext.	0.05 ± 0.005	0.04 (0.04 to 0.05)
Form 2	0.09 ± 0.066	0.06 (0.05 to 0.09)
Objet30 Prime	0.08 ± 0.009	0.08 (0.07 to 0.08)
ProJet CJP 660Pro	0.08 ± 0.018	0.08 (0.06 to 0.09)

^1^ standard deviation.

## Abbreviations

3D	Three-dimensional
ABS	Acrylonitrile butadiene styrene
AM	Additive manufacturing
ANOVA	Analysis of variance
BJ	Binder jetting
CBCT	Cone beam computed tomography
CO_2_	Carbon dioxide
CT	Computed tomography
DICOM	Digital imaging and communications in medicine
FFF	Fused filament fabrication
IPA	Isopropyl alcohol
MJ	Material jetting
PDMS	Polydimethylsiloxane
PEEK	Polyetheretherketone
PLA	Polylactic acid
PVA	Polyvinyl alcohol
RMS	Root mean square
SD	Standard deviation
SLA	Stereolithography
SLS	Selective laser sintering
STL	Standard tessellation language
UV	Ultraviolet
